# Can Early Rehabilitation Prevent Posttraumatic Osteoarthritis in the Patellofemoral Joint after Anterior Cruciate Ligament Rupture? Understanding the Pathological Features

**DOI:** 10.3390/ijms18040829

**Published:** 2017-04-14

**Authors:** Nai-Jen Chang, Ming-You Shie, Kuan-Wei Lee, Pei-Hsi Chou, Chih-Chan Lin, Chih-Jou Chu

**Affiliations:** 1 Department of Sports Medicine, Kaohsiung Medical University, Kaohsiung 807, Taiwan; alex345174@yahoo.com.tw (K.-W.L.); arthroscopy.pc@gmail.com (P.-H.C.); zhccj19@gmail.com (C.-J.C.); 2 3D Printing Medical Research Center, China Medical University Hospital, Taichung 404, Taiwan; eviltacasi@gmail.com; 3 Laboratory Animal Center, Department of Medical Research, Chi-Mei Medical Center, Tainan 710, Taiwan; jann0921@pchome.com.tw

**Keywords:** apoptosis, osteoarthritis, articular cartilage, ligament, physical therapy, histology

## Abstract

Knee instability resulting from anterior cruciate ligament (ACL) rupture is a high-risk factor for posttraumatic osteoarthritis (PTOA) in the patellofemoral joint (PFJ). However, whether non-weight-bearing and weight-bearing treatments have chondroprotective effects remains unclear. Twenty-four adult New Zealand White male rabbits were employed in this study. All animals received ACL transection in the right knee and sham surgery in the left knee. The rabbits were randomly assigned to the following groups: (I) In the sedentary (SED) group, the rabbits (*n* = 6) were simply kept in their cage; (II) In the continuous passive motion (CPM) group, the rabbits (*n* = 6) performed CPM exercise for 7 days, starting from the first postoperative day; (III) In the active treadmill exercise (TRE) group, the rabbits (*n* = 6) performed TRE for 2 weeks; (IV) In the CPM + TRE group, the rabbits (*n* = 6) executed CPM exercise, followed by TRE. Two joint surfaces (the retropatella and femoral trochlear groove) were assessed at 4 weeks after operation. Although the gross appearance in each group was comparable, histological examination revealed significant differences in the articular cartilage status. The CPM group exhibited a greater thickness of articular cartilage, maintenance of tidemark continuity, abundant glycosaminoglycan (GAG), and significantly lower inflammatory cytokine 9, e.g., tumor necrosis factor-alpha (TNF-α) 0 levels, with modest cell apoptosis (i.e., caspase-3). By contrast, the TRE group displayed the worst pathological features: an irregular cartilage surface and chondrocyte disorganization, reduced cartilage thickness, breakdown of the tidemark, depletion of collagen fibers, loss of GAG, and the highest levels of TNF-α and caspase-3 expression. Furthermore, the CPM + TRE group had more favorable outcomes than the SED group, indicating that suitable exercise is needed. The sham treatment displayed no variance in the changes in the two joint surfaces among groups. These data indicate that the application of early CPM rehabilitation is suggested for subjects in order to decrease the risk of PTOA without ACL reconstruction in the PFJ compartment in rabbits. The early TRE program, however, had harmful outcomes. Additionally, inactivity was discovered to initiate the development of PTOA.

## 1. Introduction

The knee joint includes the lower weight-bearing region of the patellofemoral joint (PFJ) and the higher weight-bearing region of tibiofemoral joint (TFJ). Patellofemoral osteoarthritis (OA) refers to the degenerative changes underneath the kneecap in the femoral trochlear groove (FTG) and retropatellar (RP) articulations, which usually result from overuse, sports injuries, patellofemoral laxity or subluxation, or imbalanced muscle strength, in addition to a joint’s inflammatory condition at the surface of the articular cartilage [1,2]. Unicompartmental arthritis of the knee generally refers to not only TFJ arthritis but also the certainly more critical PFJ arthritis [3]. Compared with the TFJ compartment, the PFJ compartment is frequently involved in the OA process [4,5]. Therefore, symptoms of knee OA are related to the PFJ compartment, and it may be more critical than the TFJ compartment. 

The anterior cruciate ligament (ACL) plays an essential role in supporting knee stability. Upon ACL rupture, an underlying cascade of corresponding biomechanical instability occurs, and in situ joint inflammation may be quickly initiated and increase cartilage degradation, subsequently leading to posttraumatic osteoarthritis (PTOA) [6,7]. To address knee instability, physical therapists strengthen knee extensors and flexors. Additionally, most orthopedic surgeons believe that ACL reconstruction prevents further instability; however, no consensus exists on whether surgery can reduce the risk of PTOA [8]. Additionally, changes in the molecular milieu of the damaged knee joint—such as greater inflammatory reactions, e.g., tumor necrosis factor-alpha (TNF-α) expression [9] and cell apoptosis (i.e., caspase-3) [10]—directly increase the development of OA. Therefore, the acute settling of balanced homeostasis in the injured joint cavity plays a decisive role in the degradation and synthesis of articular cartilage. Rehabilitation exercises, including non-weight-bearing or weight-bearing regimes, may directly manipulate in situ microenvironments in the damaged joint and are a potential chondroprotective therapy.

Continuous passive motion (CPM), a non-weight-bearing rehabilitation exercise, is recommended for the day after surgery and has been reported to reduce joint swelling and inflammatory response as well as promote tissue repair in animal models [11,12,13,14,15] and clinical practice [16,17,18]. The rationale of CPM is to promote physical stimuli in situ through passively alternating the range of motion of the joint, which generates a pumping effect and directly provides a nutritional supply of synovial fluid to the articular cartilage. Active treadmill exercise (TRE), a weight-bearing rehabilitation exercise, maintains the functional properties of the native articular cartilage and serves to recover functional activity. The fundamental rationale of active TRE rehabilitation is that it provides the mechanobiological stimulation generated during weight bearing and joint movement, which directly cultivates the articular cartilage and increases the transportation of metabolites and nutrients. The effect of TRE on cartilage has been studied in animal models of knee stability [19,20] and knee instability [21,22]. Most of the knee instability studies used an induced ACL transection (ACLT) OA model to assess the reparative effects of cell fusion therapy [23,24] or the administration of injection therapy [25,26]. 

However, few studies have evaluated whether distinct types of non-weight-bearing and weight-bearing exercise treatments or progressive combinations of such exercises might have chondroprotective effects and thereby further decrease the risk of PTOA in the PFJ. We hypothesized that early CPM rehabilitation decreases inflammatory cytokine levels and provides modest chondrocyte apoptosis as a chondroprotective effect. By contrast, the TRE regimen may not have the same effect due to a joint’s mechanical instability corresponding to the increase in extracellular matrix (ECM) depletion and chondrocyte apoptosis in the damaged PFJ. This study examined the pathological changes in the PFJ in PTOA induced through ACL rupture by using a rabbit knee instability model, wherein the subjects received early CPM and/or TRE rehabilitation.

## 2. Results

### 2.1. Rabbit Exercise Compliance and Health Status

During CPM treatment, the rabbits could passively bend their knee and easily extend their leg in a suspension position. Rabbits do not respond to verbal cues and are sometimes lazy, so all rabbits were made to perform preconditioning treadmill exercises at a low speed in a custom-designed treadmill equipped with an electrical stimulation system to adapt them to demands of the TRE regimen. After training, the rabbits could complete the required exercise with our set parameters. During the TRE program, the rabbits had no stress responses, but an abnormal gait pattern associated with knee valgus was noted. The body temperature after TRE exercise was remarkably higher than that during the resting state, indicating conditioning regulation. When the rabbits were sacrificed, no significant variation in the body weights pre- and post-surgery was noted, representing good health. The joint fluid showed no gross signs of infection but was observed to be a little yellow, mainly in the TRE group. The gross appearance of each group demonstrated comparable differences in FTG and RP articulations, with smooth articular surfaces discovered (Figure S1).

### 2.2. Histological Observations

#### 2.2.1. CPM Group Had a Significantly Greater Articular Cartilage Thickness and Maintenance of Tidemark Continuity and More Abundant Glycosaminoglycan (GAG)

**Figure 1:**
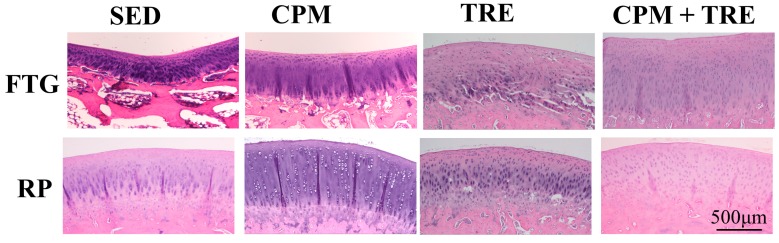
Observations of the cartilage surface and cell alignment using hematoxylin and eosin staining in the patellofemoral joint (PFJ) (the femoral trochlear groove (FTG) and retropatella (RP)) of the anterior cruciate ligament transection (ACLT) knee, in New Zealand White male rabbits. The continuous passive motion (CPM) group showed the best outcomes, with maintained smooth surfaces and cartilage thickness in addition to visible cell arrangement. However, the active treadmill exercise (TRE) group exhibited severe changes, with slightly irregular cartilage surfaces and disoriented superficial and middle zones of articular cartilage, particularly in the RP. In addition, the CPM + TRE and sedentary (SED) groups had various degrees of osteoarthritic changes in the superficial and middle zones of the articular cartilage.

**Figure 2:**
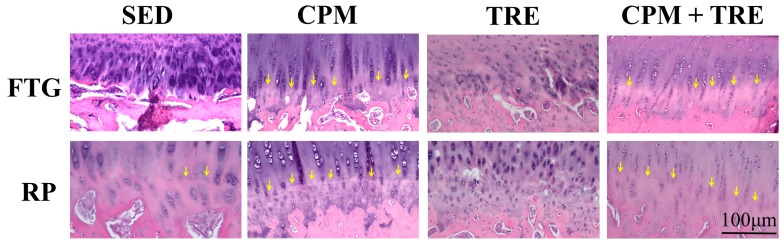
Tidemark of articular cartilage in the ACLT knees of the study groups of New Zealand White male rabbits. The CPM group exhibited maintained tidemark continuity; however, the TRE group displayed loss of tidemark continuity. Arrows indicate tidemarks.

**Figure 3:**
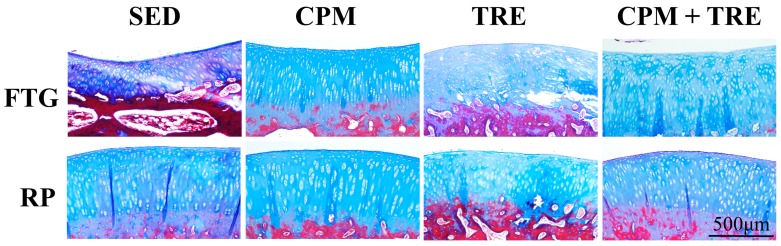
Total collagen fiber orientation, assessed using Masson’s trichrome staining in the FTG and RP of the ACLT knees of the study groups of New Zealand White male rabbits. The CPM group had sound collagen fiber orientation compared with the other groups; however, the TRE group had damaged collagen fibers, particularly in the superficial and middle zones of cartilage, suggesting early osteoarthritic features.

**Figure 4:**
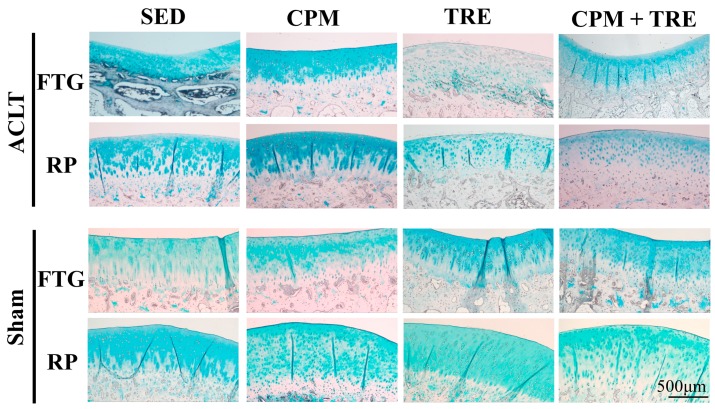
Glycosaminoglycan (GAG) distribution in the FTG and RP of the study groups of New Zealand White male rabbits, assessed using Alcian blue staining. The CPM group revealed the highest abundance of GAG. By contrast, the other groups showed varied changes, with a loss of GAG (lowest cartilage thickness) from the superficial zone to the middle zone of the articular cartilage. In addition, no changes were identified in the sham legs for all groups.

**Figure 5:**
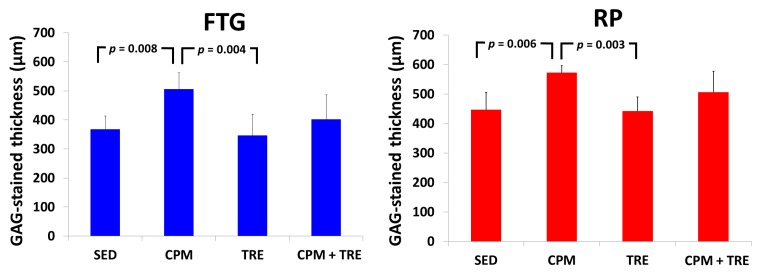
Thickness of the stained regions as an indicator of positive GAG in the ACLT knee, in New Zealand White male rabbits. Kruskal–Wallis one-way analysis of variance and post-hoc Mann–Whitney U tests were performed for between-group comparisons (*n* = 6 in each group).

Histological assessment of the articular cartilage indicated comparative degrees of deterioration in the FTG and RP from the four groups (Figure 1, Figure 2, Figure 3, Figure 4 and Figure 5). The CPM group had the best outcomes, with the maintenance of a smooth surface, adequate cartilage thickness, maintained tidemark continuity, and a visible cell arrangement in addition to abundant glycosaminoglycan (GAG; highest cartilage thickness), suggesting chondroprotective effects in the CPM group. However, the TRE group exhibited severe changes, with decreased cartilage thickness, damaged collagen fibers, and chondrocyte disorientation in the superficial and middle zones of the articular cartilage, in addition to a loss of GAG (lowest cartilage thickness). The CPM + TRE and sedentary (SED) groups showed various degrees of OA changes in the superficial and middle zones of the articular cartilage. No changes were identified in any of the compartments of the sham legs in all groups (Figure 4). 

#### 2.2.2. TNF-α and Caspase-3 Expression Increased Significantly (Highest Values) in the TRE Group and Modestly in the CPM Group

**Figure 6:**
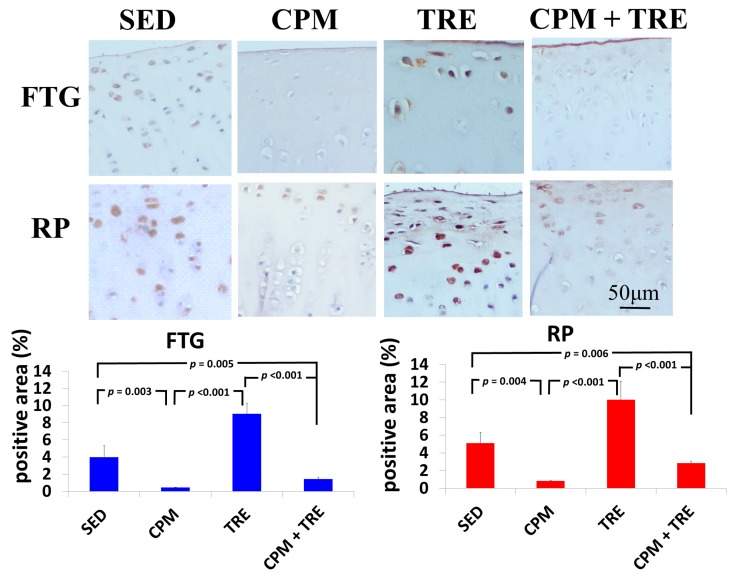
Immunohistochemical staining of tumor necrosis factor-alpha (TNF-α), in New Zealand White male rabbits. The highest significant increase in TNF-α levels was in the TRE group and the lowest in the CPM group. Kruskal–Wallis one-way analysis of variance and post-hoc Mann–Whitney U tests were performed for between-group comparisons (*n* = 6 in each group).

**Figure 7:**
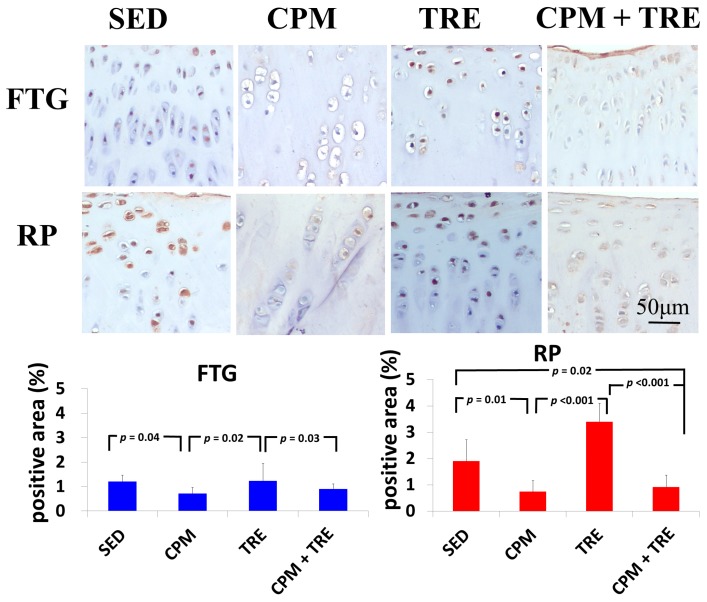
Immunohistochemical staining of caspase-3, in New Zealand White male rabbits. The highest significant increase in caspase-3 expression was in the TRE group and the lowest was in the CPM group. Kruskal–Wallis one-way analysis of variance and post-hoc Mann–Whitney U tests were performed for between-group comparisons. (*n* = 6 in each group).

To further assess specific cytokines, immunohistochemical analysis of the articular cartilage from all four groups was performed and representative images are presented in Figure 6 and Figure 7. TNF-α expression in the chondrocytes of the TRE group was significantly higher than that of the CPM and CPM + TRE groups. By contrast, the density of TNF-α in the CPM group was significantly lower (Figure 6). Furthermore, the SED group exhibited TNF-α expression, particularly in the superficial zone of the articular cartilage. With respect to the apoptosis of articular chondrocytes, the TRE group had the highest caspase-3 expression in chondrocytes in the superficial and middle zones of the articular cartilage, but the CPM group had modest expression. Furthermore, the SED group displayed caspase-3 expression in the superficial and middle zones of the articular cartilage, particularly on the RP surface (Figure 7).

## 3. Discussion 

This is the first study to provide an understanding of the pathological changes in PTOA induced through ACL rupture in the PFJ. This was achieved using a rabbit knee instability model wherein subjects received early CPM and/or TRE rehabilitation. The exercises generated modulation and redistributed the microenvironments in the knee joint space after ACL rupture. Most importantly, we demonstrated that early CPM may have implications for potential chondroprotective management and the prevention of PTOA in the PFJ. The CPM group had smooth cartilage surfaces, maintained tidemark continuity, and had oriented chondrocytes, along with an enriched synthesis of GAG, modest inflammatory cytokine (i.e., TNF-α) levels, and apoptosis of articular chondrocytes (i.e., caspase-3; Figure 1, Figure 2, Figure 3, Figure 4, Figure 5, Figure 6 and Figure 7). By contrast, the other treatment groups displayed variable chondrocyte disorientation in the superficial and middle zones of articular cartilage (Figure 1) and a loss of GAG (thin cartilage thickness; Figure 4 and Figure 5) as well as augmented expression of TNF-α and caspase-3 (Figure 6 and Figure 7). The observed potential chondroprotective effects may be attributable to the reasonable exercise program and the presence of an appropriate growth microenvironment associated with reduced inflammatory cytokines in the reparative site. 

Suitable rehabilitation exercise for knee joints is needed for the regulation of cartilage turnover. Benefits include improvements in collagen formation, GAG expression, and bone remodeling [20,27,28]. However, the optimal parameters—including intervention timing, intensity, duration, frequency, and type of exercise—are still unknown [16,29,30]. Performance of weight-bearing exercise too early could damage the native cartilage due to joint instability or imbalanced muscle strength, whereas initiation of exercise too late could have no beneficial effects due to an insufficient growth microenvironment [20]. In the present study, the rationale for the CPM, TRE, and combination time frames was according to our previous findings and experiences in clinical practice. In clinical practice, lower weight–bearing exercise is first recommended, with progress to weight-bearing exercises performed during rehabilitation programs for knee injury [30]. Similarly, the current study was also designed based on the standard protocol for rehabilitation. Briefly, CPM was first performed, followed by TRE. Early CPM has been recommended for shortly after joint damage/effusion has occurred. In this study, we adopted a CPM program comprising exercise of 15 min/day for 7 days/week, starting on the day following surgery. In physical rehabilitation protocols, 15–20-min passive motion treatments with the assistance of a physical therapist are frequently incorporated after knee surgery [31]. Accordingly, given practical considerations in animal CPM application, we used 15-min short-term stimuli in the knee joints to minimize the stress to the rabbits instead of 8 h/day [32] or 24 h/day [33] therapies. In addition, our previous study indicated that immediate CPM rehabilitation for 15 min/day enhances cartilage regeneration for the repair of osteochondral defects in rabbits [12]. In the present study, CPM use had a chondroprotective effect on knee instability in rabbits. Regarding active weight-bearing exercises, our previous study demonstrated that—in rabbits with an osteochondral defect in the PFJ and that performed early treadmill exercise for 15 min/day, 5 days/week, for a period of 2 sequential weeks—in situ endogenous growth factor (i.e., TGF-β1) expression and anti-inflammatory effects (e.g., TNF-α expression) were induced in the reparative site [28]. Nonetheless, this treatment effect exhibited harmful outcomes in the ACL rupture model. Knee instability induced by the ACLT increased tibiofemoral anterior translation and subsequently increased the shear and compressive forces on the RP and FTG interface in the PFJ. Eventually, prolonged alteration in the kinematics caused early degenerative changes, as previously described [1]. In the present study, although the gross appearance did not change visibly, histological examination revealed significant differences in the articular cartilage status. We observed chondrocyte disorganization, ECM depletion corresponding to the loss of GAG from the superficial to the middle zones of articular cartilage, and tidemark breakdown (Figure 1, Figure 2, Figure 3 and Figure 4). In particular, the tidemark is firmly connected to the upper cartilage and underlying subchondral bone, and the interaction between the cartilage and remodeled bone is crucial for the regulation of cartilage turnover [34]. Once the continuity of the tidemark is destroyed (e.g., through large mechanical stresses), the marked irregularity or delamination of the osteochondral junction consequently leads to full thickness cartilage degeneration [35], further causing varying degrees of PTOA.

An appropriate growth microenvironment may provide a niche to prevent the development of PTOA in the PFJ. This microenvironment can be directly affected by CPM rehabilitation exercise or active joint movement (i.e., TRE) used as preventive managements. CPM involves a passive pumping effect [14], which facilitates a squeeze in-and-out movement of the synovial fluid through alternative knee flexion and extension, subsequently promoting the clearance of inflammatory cytokines and redistributing the microenvironments in the damaged joint cavity. In addition, active TRE rehabilitation provides mechanobiological stimulation generated during weight bearing and joint movement, which directly cultivates the articular cartilage and improves the transportation of metabolites and nutrients. Numerous investigations using knee stability models have demonstrated that CPM [11,12,33,36,37] or TRE [19,20] rehabilitation can benefit articular cartilage growth. In the present study, we examined the knee instability induced by ACLT and assessed the effects of the rehabilitation exercises. The CPM group exhibited the best chondroprotective outcomes; however, the TRE group experienced the worst outcomes, as evidenced by their thinnest cartilage thickness, chondrocyte disorientation, loss of GAG, and highest expression of inflammatory and apoptosis markers (Figure 1, Figure 2, Figure 3, Figure 4, Figure 5, Figure 6 and Figure 7). Therefore, the roles played by non-weight-bearing and weight-bearing exercises in the change of microenvironments during early healing should be considered. The causative mechanism for PTOA remains unclear; however, it may be caused by ECM depletion and complex catabolic cytokine changes. Rehabilitation exercises were potential mediators in the present study. Compared with the other treatments, the application of CPM successfully prevented the occurrence of PTOA and also sustained GAG. This suggests that CPM has an anti-inflammatory effect on damaged knee joints. This net effect can thus reduce inflammatory markers (i.e., TNF-α expression), maintain chondrocyte health (e.g., modest apoptosis of articular chondrocytes), and retain ECM. Conversely, extra weight-bearing rehabilitation exercise in the early knee instability stage induced by ACLT, potentiates harmful effects. These effects are augmented significantly by the catabolic cytokine TNF-α, elevated expression of caspase-3 (Figure 6 and Figure 7), and depletion of the GAG content from the superficial to middle zone layers (Figure 4), indicating that this activates cartilage degeneration. Furthermore, because of physical inactivity or nonreceiving of active rehabilitation (e.g., SED group), posttraumatic hemarthrosis in response to inflammation storage persists in the joint cavity [38], resulting in inadequate refreshment of the joint fluid, which triggers a catabolic reaction in the joint and ultimately causes cartilage degeneration.

Future studies are warranted for understanding the causes of PTOA after ACL rupture. First, inflammatory factors in the synovial fluid should be addressed to assess the whole joint microenvironment status. Second, gait analysis and the mechanical properties of the articular cartilage should be further investigated. Third, the long-term outcomes should be determined to assess the substantial chondroprotective effects, given that the current data indicate promising outcomes. Fourth, the pathological changes between ACL reconstruction and non-ACL reconstruction should be compared. Fifth, larger preclinical animal trials are needed prior to clinical application. In summary, the current data provide an understanding of the pathological changes in PTOA in the PFJ compartment after ACL rupture and after physical rehabilitation exercises. The studied early exercise strategy may have implications for potential chondroprotective interventions in rehabilitation regimens at the time of injury and for preventing PTOA in the PFJ compartment.

## 4. Materials and Methods 

### 4.1. An ACLT Model

All surgical animal experiments and aseptic procedures were approved by the Animal Care and Use Committee (approval no 104071707). Twenty-four 5–6-month-old New Zealand White male rabbits weighing 3.0–3.5 kg were used in this study (Figure 1). Before surgery, general anesthesia was induced via a subcutaneous injection of Zoletil 50 (25 mg/kg; Virbac, Carros, France) and was maintained through the automatic ventilator administration of a mixture of 2% isoflurane (Panion & BF Biotech Inc., Taipei, Taiwan) and oxygen/nitrous oxide (1/0.4 L/min). Under general anesthesia, the rabbit’s bilateral legs were shaved, brushed, and disinfected using 1% ethanoliodine. An ACLT was performed in the rabbit knee joint model. The joint capsule from the right knee was opened through an anteromedial parapatellar longitudinal incision. The patella was dislocated laterally. Subsequently, the knee joint was maximally flexed to expose the ACL. The ACL was then transected with a blade. The patella was repositioned in the normal patellar position, followed by wound closure. Joint instability was assessed in all ACLT knees with positive Lachman and anterior drawer tests. In the sham leg, the left knee was opened as previously described, but an ACLT was not performed. The joint capsule was closed using 3-0 absorbable Vicryl sutures. The subcutaneous tissues and skin wound were repaired using 3-0 nylon sutures. Each rabbit was individually housed in a stainless steel cage. An antibiotic (25 mg/kg, enrofloxacin) was administered during the first 3 days after surgery. The skin wound areas were dressed with povidone iodine for 7 days. Data regarding body weight, food and drink, urination, wound healing, and functional activity after surgery were recorded for all rabbits.

### 4.2. Treatment Regimens

To adapt them to the required exercise, all rabbits executed preconditioning treadmill exercises (progressive to 10 min/day, 5 days/week) before surgery. Subsequently, the rabbits (six rabbits in each group) were randomly distributed into one of four rehabilitation groups: the SED, CPM, TRE, and CPM + TRE groups. (I) In the SED group, the rabbits were kept singly in their cages without any further exercise until sacrifice. (II) In the CPM group, passive exercise using an electrical motor and gear apparatus was applied to the rabbit knee without anesthesia on the day following surgery. The rehabilitation conditions were set to adjustable knee flexion/extension for 15 min/day for 7 consecutive days, as previously described [12]. After CPM treatment, the rabbits were housed and allowed free cage activity, with restricted weight-bearing activity. (III) In the TRE group, the rabbits were managed with early active weight-bearing exercise on the treadmill at 2 weeks after surgery, for 17 m/min, 5 days/week, for a period of 2 sequential weeks, as previously described [28]. After TRE treatment, the rabbits were returned to their cage. (IV) In the CPM+TRE group, the rabbits were managed with CPM for 7 days after surgery, housed in a cage for the following 2 weeks, and then made to perform TRE for 2 weeks, as per the aforementioned protocol. All rabbits were sacrificed at 4 weeks after surgery, because previous investigations have proposed that a 4-week ACLT is adequate to successfully induce the development of OA in rabbits [21].

### 4.3. Histological Processing and OA Scores

After euthanasia at 4 weeks post-operation, the knees were assessed in two regions: FTG and RP. All histological sections were performed by the Department of Pathology of Chi-Mei Medical Center. The resected knees were fixed for histological evaluation in neutral-buffered formalin, dehydrated in graded alcohol, decalcified, sectioned perpendicular to the longitudinal axis, infiltrated, and embedded in paraffin wax, according to standard processing protocol. Four-micron-thick sections were stained with hematoxylin and eosin for cartilage surface and cell morphological assessments, Alcian blue for GAG distribution determination, and Masson’s trichrome for general collagen orientation evaluation. The sections were observed under a light microscope (Olympus CX51, Tokyo, Japan) and recorded using a digital CCD camera (Tucsen, Truechrome Metrics, Puchheim, Germany). Additionally, the thicknesses of the stained regions indicating positive GAG in the ACLT knees were measured using Image-Pro Plus software 7.0 (Media Cybernetics Inc., Rockville, MD, USA). Furthermore, specific cytokines such as TNF-α (inflammatory response) and caspase-3 (apoptosis of articular chondrocytes) in the tissues were assessed through immunohistochemical staining of the sections. Endogenous peroxidase was treated with a peroxidase-blocking reagent included in the rabbit/mouse HRP/DAB detection system (BioTnA Inc., Kaohsiung, Taiwan) kit. All primary antibodies (dilution factor 1:200) were treated at room temperature. All staining protocols followed the suppliers’ guidelines. A positive signal was recognized as a brown precipitate using DAB substrate. The sections were counterstained with hematoxylin, dehydrated, and coverslipped. Furthermore, for each knee, the proportions of the total area of the articular cartilage that was positive for TNF-α or caspase-3 were calculated using the Image-Pro Plus software 7.0. The scoring/image acquisition was assessed blindly.

### 4.4. Statistics

The results are reported as mean ± 95% confidence interval. SPSS 17.0 (SPSS Inc., Chicago, IL, USA) was used for statistical analysis. Nonparametric analysis was performed to analyze the data, owing to the non-normally distributed data obtained through the Shapiro–Wilk test in addition to the nonhomogeneity of variance data obtained through Levene’s test. Kruskal–Wallis one-way analysis of variance and post-hoc Mann–Whitney U tests were performed for between-group comparisons. *p* < 0.05 was considered statistically significant.

## 5. Conclusions

Early CPM rehabilitation is suggested to decrease the risk of PTOA without ACL reconstruction in the PFJ compartment in rabbits. Use of early TRE, however, had harmful outcomes. Furthermore, nonreceiving of active rehabilitation initiated the development of PTOA.

## Supplementary Materials

Supplementary materials can be found at www.mdpi.com/1422-0067/18/4/829/s1.

## Abbreviations

PFJ	Patellofemoral joint
TFJ	Tibiofemoral joint
FTG	Femoral trochlear groove
RP	Retropatella
PTOA	Posttraumatic osteoarthritis
ACL	Anterior cruciate ligament
TNF-α	Tumor necrosis factor-alpha
CPM	Continuous passive motion
TRE	Treadmill exercise
ROM	Range of motion
ACLT	Anterior cruciate ligament transection
GAG	Glycosaminoglycan
