# Supplementary material for: Can Early Rehabilitation Prevent Posttraumatic Osteoarthritis in the Patellofemoral Joint after Anterior Cruciate Ligament Rupture? Understanding the Pathological Features
Source: Int J Mol Sci. 2017 Apr 14;18(4):829. doi: 10.3390/ijms18040829 (PMC5412413; doi:10.3390/ijms18040829)
Supplement: Supplementary file 1 [file ijms-18-00829-s001.pdf]

Supplementary Materials: Can Early Rehabilitation Prevent Posttraumatic Osteoarthritis in the Patellofemoral Joint after Anterior Cruciate Ligament Rupture? Understanding the Pathological Features

Nai-Jen Chang, Ming-You Shie, Kuan-Wei Lee, Pei-Hsi Chou, Chih-Chan Lin and Chih-Jou Chu

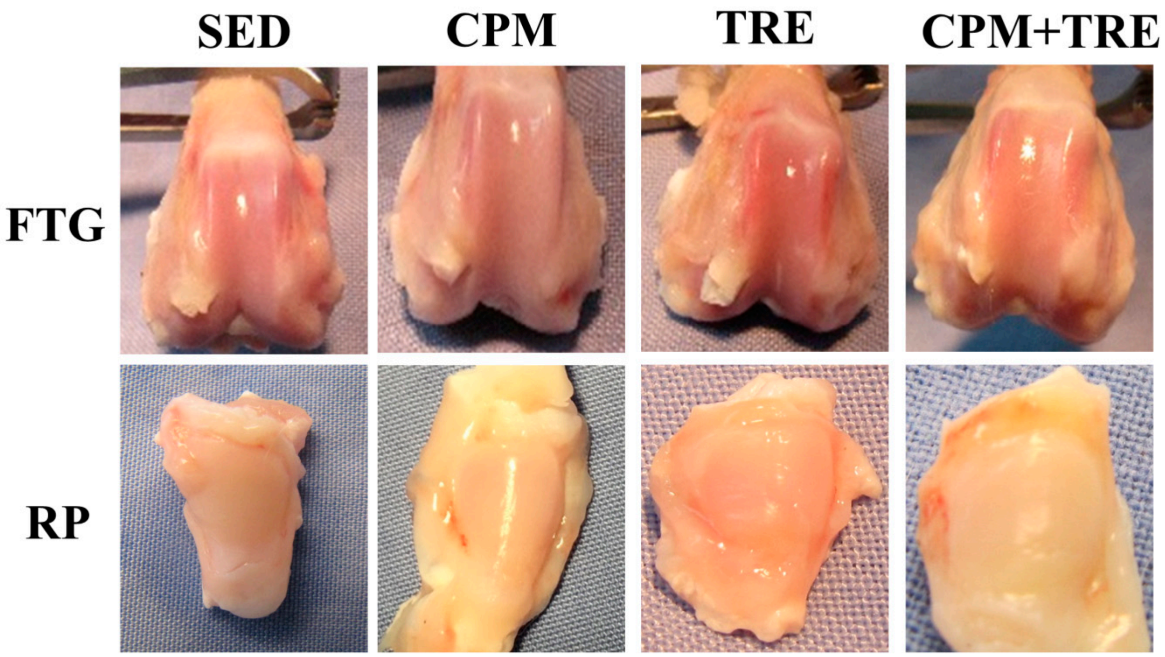

Figure S1. Evaluation of the gross appearance in the ACL transection (ACLT) knee.
